# A randomized, open-label, Phase III study of obinutuzumab or rituximab plus CHOP in patients with previously untreated diffuse large B-Cell lymphoma: final analysis of GOYA

**DOI:** 10.1186/s13045-020-00900-7

**Published:** 2020-06-06

**Authors:** Laurie H. Sehn, Maurizio Martelli, Marek Trněný, Wenxin Liu, Christopher R. Bolen, Andrea Knapp, Deniz Sahin, Gila Sellam, Umberto Vitolo

**Affiliations:** 1 BC Cancer Centre for Lymphoid Cancer and the University of British Columbia, Vancouver, BC Canada; 2 Department of Translational and Precision Medicine, Sapienza University of Rome, Rome, Italy; 3 Charles University General Hospital, Prague, Czech Republic; 4 Roche Pharma Development, Shanghai, China; 5 Genentech, Inc., South San Francisco, CA USA; 6 F. Hoffmann-La Roche Ltd, Basel, Switzerland; 7 Candiolo Cancer Institute, FPO-IRCCS, (Turin), Candiolo, Italy

**Keywords:** Diffuse large B cell lymphoma, Obinutuzumab, Rituximab, Immunochemotherapy, Outcomes

## Abstract

**Background:**

Rituximab (R) plus cyclophosphamide, doxorubicin, vincristine, and prednisone (CHOP) is the current standard therapy for diffuse large B cell lymphoma (DLBCL). Obinutuzumab (G), a glycoengineered, type II anti-CD20 monoclonal antibody, has shown activity and an acceptable safety profile when combined with CHOP (G-CHOP) in patients with advanced DLBCL. We present the final analysis results of the Phase III GOYA study (NCT01287741), which compared the efficacy and safety of G-CHOP versus R-CHOP in patients with previously untreated DLBCL.

**Methods:**

Patients aged ≥ 18 years with previously untreated advanced DLBCL were randomly assigned to receive eight 21-day cycles of R or G, plus six or eight cycles of CHOP. The primary endpoint was investigator-assessed progression-free survival (PFS). Secondary endpoints included overall survival, other time-to-event endpoints, and safety; investigator-assessed PFS by cell of origin subgroup was an exploratory endpoint.

**Results:**

A total of 1418 patients were randomized, with 1414 included in this final analysis (G-CHOP, *N* = 704; R-CHOP, *N* = 710). Five-year PFS rates were 63.8% and 62.6% for G-CHOP and R-CHOP, respectively (stratified hazard ratio 0.94, 95% CI 0.78–1.12; *p* = 0.48). The results of the secondary efficacy endpoints did not show a benefit of G-CHOP over R-CHOP. In the exploratory analysis, a trend towards benefit with G-CHOP over R-CHOP was apparent in the patients with germinal center B cell DLBCL. The safety profile of G-CHOP was as expected, and no new safety signals were observed. More grade 3–5 (75.1% vs 65.8%), serious (44.4% vs 38.4%), and fatal (6.1% vs 4.4%) adverse events (AEs) were observed in the G-CHOP arm compared with the R-CHOP arm, respectively, with the most common fatal AEs being infections. A higher incidence of late-onset neutropenia occurred in the G-CHOP arm (8.7%) versus the R-CHOP arm (4.9%).

**Conclusions:**

The final analysis, similar to the primary analysis, did not show a PFS benefit of G-CHOP over R-CHOP in previously untreated patients with DLBCL. The results of the secondary endpoints were consistent with the primary endpoint. Further exploratory analyses and investigation of biomarkers are ongoing.

## Background

Rituximab (R) in combination with cyclophosphamide, doxorubicin, vincristine, and prednisone (CHOP) is the current standard of care for diffuse large B cell lymphoma (DLBCL) [1–3]. Despite treatment with R-CHOP, many patients’ relapse and outcomes remain poor with salvage therapies [4]. Obinutuzumab (GA101; G) is a fully humanized, glycoengineered, type II anti-CD20 monoclonal antibody which has shown greater direct cell death induction, antibody-dependent cellular cytotoxicity, and antibody-dependent cellular phagocytosis than R [5–8].

In a Phase II study in patients with advanced DLBCL, G demonstrated promising activity and an acceptable safety profile when combined with CHOP (G-CHOP) as a first-line treatment [9]. GOYA (NCT01287741) was a randomized, open-label, multicenter Phase III study that evaluated the efficacy and safety of G-CHOP compared with R-CHOP in patients with previously untreated DLBCL. Results from the primary analysis (clinical cut-off date, 29 April 2016), which had a median observation period of 29 months, showed that G-CHOP did not significantly improve investigator-assessed progression-free survival (PFS) compared with R-CHOP [10]. Here, we present the updated results from the final analysis of GOYA.

## Methods

The study design and methodology of the GOYA study are described in full elsewhere [10]. In brief, patients were included if they were at least 18 years of age and had histologically documented, previously untreated, CD20-positive DLBCL; adequate hematologic function; ≥ 1 bi-dimensionally measurable lesion; an Eastern Cooperative Oncology Group (ECOG) performance status of ≤ 2; and an International Prognostic Index (IPI) risk group of high, high-intermediate, or low-intermediate risk. Low-risk patients with an IPI score of 1 (not due to age alone) or 0 with bulky disease (1 lesion ≥ 7.5 cm) were also eligible. Using stratified permuted block randomization, patients were randomized 1:1 to eight (21 days) cycles of G (1000 mg intravenous injection [IV] on days 1, 8 and 15, cycle 1 and day 1, cycles 2–8) or R (375 mg/m^2^ IV on day 1 of cycles 1–8) in combination with six or eight cycles of CHOP. The randomization stratification factors were number of planned cycles of CHOP, IPI score, and geographic region. Pre-planned radiotherapy was allowed for bulky or extranodal disease. The primary endpoint was investigator-assessed PFS. Secondary endpoints included independent review committee (IRC)-assessed PFS (primary analysis only); overall survival (OS); complete response (CR) and overall response rate (ORR) according to the modified Cheson 2007 criteria [11] by computed tomography (CT) and CT incorporating positron emission tomography (PET); event-free survival (EFS; defined as the period from the date of randomization until the date of disease progression, relapse, initiation of a new non-protocol-specified anti-lymphoma treatment, or death from any cause); disease-free survival (DFS; defined as the percentage of patients with CR at the end of treatment); time to next anti-lymphoma treatment; and safety. An exploratory endpoint of investigator-assessed PFS according to cell of origin (COO; germinal center B cell [GCB] or activated B cell [ABC]) based on gene expression profiling using the NanoString Research-Use-Only assay (NanoString Technologies, Inc., Seattle, WA) [12, 13] was also analyzed.

### Statistical analysis

As previously described, it was planned to enroll approximately 1400 patients over 3 years, which was expected to yield 405 PFS events for the primary analysis [10]. The final analysis was conducted once patients had completed at least 3 years of follow-up. Comparisons between treatment arms for time-to-event endpoints were performed using a stratified two-sided log-rank test (*α* = 0.05). The analyses stratification factors were number of planned cycles of CHOP (6 or 8) and IPI score. Kaplan–Meier analysis was also used to analyze time-to-event endpoints. Estimates of treatment effect were calculated using Cox proportional hazards regression and are presented as stratified hazard ratios (HR) with 95% confidence intervals (CI).

## Results

### Patient characteristics and treatment

A total of 1418 patients were enrolled across 207 sites in 29 countries between July 2011 and June 2014. Of these, 1414 patients were included in the final analysis (clinical cut-off date, 31 January 2018); four patients from a single study site were excluded due to serious Good Clinical Practice non-compliance. A total of 704 and 710 patients were included in the G-CHOP and R-CHOP arms, respectively (intent-to-treat population), with 702 and 701 receiving at least one dose of study treatment (safety population). Demographic and baseline characteristics were well balanced between the two groups (Table 1). COO data were available for 933 patients, and the distribution of patients with each DLBCL subtype was similar between treatment arms. In total, the median time from diagnosis to randomization was 24.0 days (range, 1.0–1104.9) (G-CHOP 23.1 days [range, 3.0–1104.9]; R-CHOP: 25.0 days [range, 1.0–264.8]).

**Table 1:** Baseline patient and disease characteristics (intent-to-treat population)

Characteristic	R-CHOP (*N* = 710)	G-CHOP (*N* = 704)
Median age (range), years	62.0 (18–83)	62.0 (18–86)
Male sex, *n* (%)	382 (53.8)	368 (52.3)
Geographic region, *n* (%)		
Eastern Europe	99 (13.9)	97 (13.8)
Western Europe	215 (30.3)	211 (30.0)
Central and South America	19 (2.7)	13 (1.8)
North America	107 (15.1)	109 (15.5)
Asia	256 (36.1)	258 (36.6)
Other	14 (2.0)	16 (2.3)
ECOG PS, *n* (%)^a^		
0–1	611 (86.1)	617 (87.8)
2	99 (13.9)	86 (12.2)
Ann Arbor stage, *n* (%)^b^		
I and II	171 (24.1)	169 (24.0)
III and IV	538 (75.9)	535 (76.0)
IPI risk group, *n* (%)		
Low/low-intermediate	408 (57.5)	374 (53.1)
High-intermediate	192 (27.0)	220 (31.3)
High	110 (15.5)	110 (15.6)
No. of planned CHOP cycles, *n* (%)		
6	524 (73.8)	521 (74.0)
8	186 (26.2)	183 (26.0)
LDH elevated, *n*(%)^c^		
Yes	403 (57.1)	415 (59.0)
Extranodal involvement, *n* (%)		
Yes	466 (65.6)	484 (68.8)
Bulky disease (≥ 7.5 cm), *n* (%)^d^	262 (37.0)	261 (37.2)
Median time from diagnosis to randomization (range), days^e^	25.0 (1.0–264.8)	23.1 (3.0–1104.9)
Cell of origin, *n* (%)^f^		
GCB	269 (58.2)	271 (57.5)
ABC	118 (25.5)	125 (26.5)
Unclassified	75 (16.2)	75 (15.9)

*ABC* activated B cell, *ECOG PS* Eastern Cooperative Oncology Group performance status, *GCB* germinal center B cell, *G-CHOP* obinutuzumab plus cyclophosphamide, doxorubicin, vincristine, and prednisone, *IPI* International Prognostic Index, *LDH* lactate dehydrogenase, *R-CHOP* rituximab plus cyclophosphamide, doxorubicin, vincristine, and prednisone

^a^*N* = 710 for R-CHOP and *N* = 703 for G-CHOP

^b^*N* = 709 for R-CHOP and *N* = 704 for G-CHOP

^c^*N* = 706 for R-CHOP and *N* = 703 for G-CHOP

^d^*N* = 708 for R-CHOP and *N* = 701 for G-CHOP

^e^*N* = 708 for R-CHOP and *N* = 700 for G-CHOP

^f^*N* = 462 for R-CHOP and *N* = 471 for G-CHOP

**Fig. 1:**
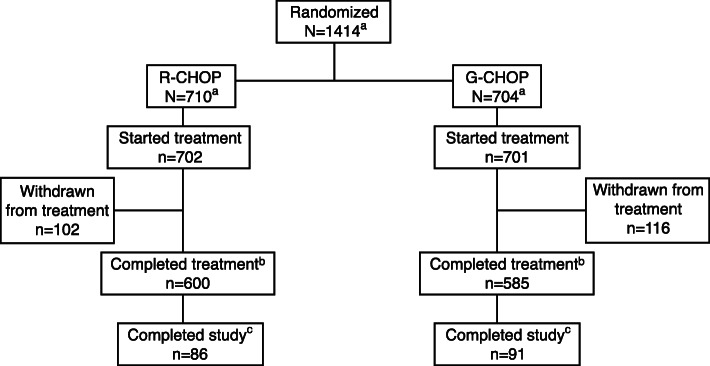
Patient disposition from the final analysis of the GOYA trial. ^a^Overall, 1418 patients were randomized (G-CHOP arm: *N* = 706 and R-CHOP arm: *N* = 712) to the study; however, due to a serious Good Clinical Practice non-compliance at a single site, data from all 4 patients enrolled at the site (two patients in each arm) were excluded from the final analysis. ^b^A patient was considered to have completed the treatment if they received all 8 cycles of study treatment. ^c^The end of the study was defined as the last patient’s last visit and occurred at approximately 6.5 years (78 months) after the first patient was enrolled to allow all patients to have ≥ 3 years of follow-up post-treatment. *G-CHOP,* obinutuzumab plus cyclophosphamide, doxorubicin, vincristine, and prednisone; *R-CHOP*, rituximab plus cyclophosphamide, doxorubicin, vincristine, and prednisone

A total of 117 patients (16.7%) in the G-CHOP arm and 105 patients (15.0%) in the R-CHOP arm discontinued any component of study treatment; 116 patients (16.5%) in the G-CHOP and 102 patients (14.5%) in the R-CHOP arm discontinued antibody treatment. The most common reason for antibody treatment discontinuation was adverse events (AEs), with a higher percentage of patients in the G-CHOP arm than in the R-CHOP arm discontinuing antibody treatment due to an AE (10.4% vs 6.1%) (Fig. 1).

New (unplanned) anti-lymphoma treatment (including systemic new anti-lymphoma treatment, radiotherapy, or surgical procedure) was received by 113 patients (53 and 60 in the G-CHOP and R-CHOP arms, respectively) prior to disease progression and by 261 patients (122 and 139 in the G-CHOP and R-CHOP arm, respectively) after disease progression.

### Efficacy

**Fig. 2:**
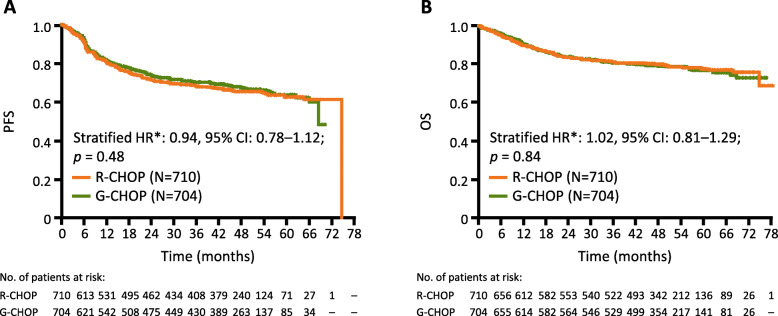
Kaplan–Meier estimates of PFS by treatment group and OS by treatment group. **a** Investigator-assessed PFS (primary endpoint) by treatment (ITT population), in which no significant difference was found for G-CHOP compared with R-CHOP. **b** OS by treatment (ITT population), which showed no significant difference in survival between treatment groups. *Stratified by planned number of CHOP cycles and IPI score. *CI*, confidence interval; *G-CHOP*, obinutuzumab plus cyclophosphamide, doxorubicin, vincristine, and prednisone; *ITT*, intent-to-treat; HR, hazard ratio; PFS, progression-free survival; *OS*, overall survival; *R-CHOP*, rituximab plus cyclophosphamide, doxorubicin, vincristine, and prednisone

At the time of final analysis (median observation time, 48.0 months; range, 0.1–76.5 months for G-CHOP and 47.4 months; range, 0.1–78.2 months for R-CHOP), 224 (31.8%) patients and 233 (32.8%) patients had experienced an investigator-assessed PFS event in the G-CHOP and R-CHOP arms, respectively (stratified HR 0.94, 95% CI 0.78–1.12; *p* = 0.48) (Table 2, Fig. 2). Estimated 5-year PFS rates were 63.8% and 62.6% for G-CHOP and R-CHOP, respectively. The results of the secondary endpoints were similar between the two treatment arms (Table 2). In total, 149 (21.2%) patients in the G-CHOP arm and 145 (20.4%) patients in the R-CHOP arm had an OS event. Estimated 5-year OS rates were 77.0% and 77.7% for G-CHOP and R-CHOP, respectively (stratified HR 1.02, 95% CI 0.81–1.29; *p* = 0.84; Fig. 2). EFS at 5 years was 60.6% and 58.9% in the G-CHOP arm and R-CHOP arm, respectively (stratified HR 0.95, 95% CI 0.80–1.12; *p* = 0.53). Time to start of new anti-lymphoma treatment was similar between the two groups, with events experienced by 33.8% of patients in the G-CHOP arm and 35.2% of patients in the R-CHOP arm (stratified HR 0.93, 95% CI 0.78–1.12; *p* = 0.45). The proportion of patients with a CR was similar for the G-CHOP and R-CHOP arms when assessed with CT incorporating PET or CT alone (56.5% vs 59.1% and 35.4% vs 33.9%, respectively). The ORR was also similar between treatment arms with CT incorporating PET or CT alone (77.1% vs 77.6% and 81.4% vs 80.1% for G-CHOP vs R-CHOP, respectively).

**Table 2:** Summary of efficacy endpoints (intent-to-treat population)

		Investigator assessment	
Endpoint	R-CHOP (*N* = 710)		G-CHOP (*N* = 704)
Median observation time (range), months	47.4 (0.1–78.2)		48.0 (0.1–76.5)
Investigator-assessed PFS (primary endpoint)			
Patients with event, *n* (%)	233 (32.8)		224 (31.8)
5-year PFS, % (95% CI)	62.6 (58.1–66.8)		63.8 (59.3–68.0)
Stratified HR (95% CI)		0.94 (0.78–1.12)	
*P* (log-rank)*		*P* = 0.48	
OS			
Patients with event, *n* (%)	145 (20.4)		149 (21.2)
5-year OS, % (95% CI)	77.7 (74.1–80.9)		77.0 (73.3–80.3)
Stratified HR (95% CI)		1.02 (0.81–1.29)	
*P* (log-rank)*		*P* = 0.84	
DFS in patients with investigator-assessed CR			
Patients with event, *n* (%)	78 (19.8)		93 (22.3)
Stratified HR (95% CI)*		1.19 (0.88–1.61)	
Investigator-assessed EFS			
Patients with event, *n* (%)	265 (37.3)		257 (36.5)
Proportion of EFS at 5 years, % (95% CI)	58.9 (54.5–63.1)		60.6 (56.3–64.6)
Stratified HR (95% CI)		0.95 (0.80–1.12)	
*P* (log-rank)*		*P* = 0.53	
Time to start of new anti-lymphoma treatment			
Patients with event, *n* (%)	250 (35.2)		238 (33.8)
Stratified HR (95% CI)		0.93 (0.78–1.12)	
*P* (log-rank)*		*P* = 0.45	
Investigator-assessed response rate (CT with PET) at end of treatment^a^	**R-CHOP (*N*= 665)**		**G-CHOP (*N*= 669)**
ORR			
*n* (%)	516 (77.6)		516 (77.1)
Percentage difference (95% CI)		− 0.46 (− 5.03–4.11)	
CR rate			
*n* (%)	393 (59.1)		378 (56.5)
Percentage difference (95% CI)		− 2.60 (− 7.97–2.78)	
Investigator-assessed response rate (CT without PET) at end of treatment^a^	**R-CHOP (*N*= 710)**		**G-CHOP (*N*= 704)**
ORR			
*n* (%)	569 (80.1)		573 (81.4)
Percentage difference (95% CI)		1.25 (− 2.93–5.43)	
CR rate			
*n* (%)	241 (33.9)		249 (35.4)
Percentage difference (95% CI)		1.43 (− 3.61–6.46)	

*CR* complete response, *DFS* disease-free survival, *EFS* event-free survival, *G-CHOP* obinutuzumab plus cyclophosphamide, doxorubicin, vincristine, and prednisone, *HR* hazard ratio, *ORR* overall response rate, *OS* overall survival, *PET* positron emission tomography, *PFS* progression-free survival, *R-CHOP* rituximab plus cyclophosphamide, doxorubicin, vincristine, and prednisone

*Stratification factors were International Prognostic Index and planned number of CHOP cycles (6 or 8)

^a^According to the revised response criteria

**Fig. 3:**
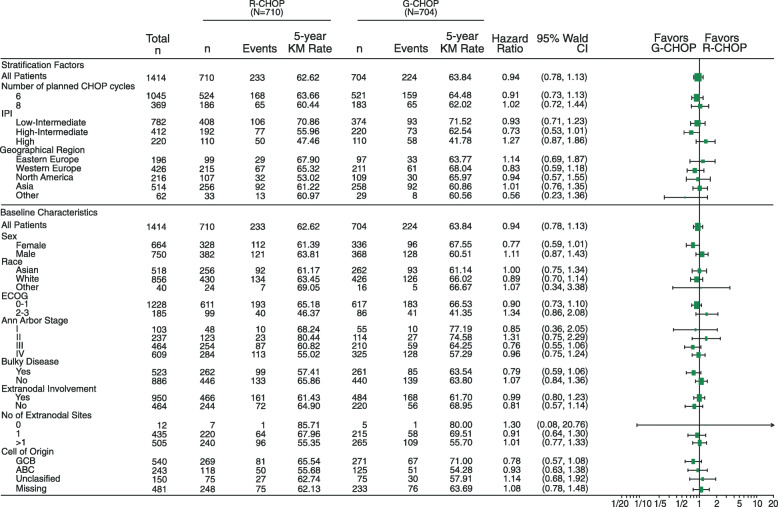
Forest plot of unstratified HRs for investigator-assessed PFS by treatment group and patient subgroup. *CHOP*, cyclophosphamide, doxorubicin, vincristine, and prednisone; *CI*, confidence interval; *ECOG PS*, Eastern Cooperative Oncology Group performance status; *G-CHOP*, obinutuzumab plus cyclophosphamide, doxorubicin, vincristine, and prednisone; *HR*, hazard ratio; *IPI*, International Prognostic Index; *KM*, Kaplan–Meier; *R-CHOP*, rituximab plus cyclophosphamide, doxorubicin, vincristine, and prednisone

Overall, the results of the PFS subgroup analyses were consistent with PFS in the overall population with no significant difference observed between treatment arms for any subgroup according to stratification factors and baseline characteristics (Fig. 3); although, notably, patients with a high IPI score at baseline trended towards a better response to treatment with R-CHOP compared with G-CHOP (low-intermediate: HR 0.93, 95% CI 0.71–1.23; high-intermediate: HR 0.73, 95% CI 0.53–1.01; high: HR 1.27, 95% CI 0.87–1.86; Fig. 3).

**Fig. 4:**
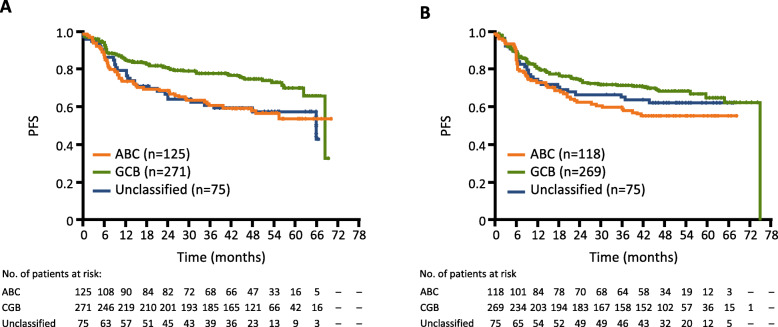
Kaplan–Meier estimates of PFS by COO status by treatment group. **a** Investigator-assessed PFS by COO with G-CHOP, where GCB appeared to have a better outcome compared with ABC and unclassified subgroups. **b** Investigator-assessed PFS by COO with R-CHOP, where GCB also appeared to have a better outcome versus ABC and unclassified subgroups. *ABC*, activated B cell; *COO*, cell of origin; *GCB*, germinal center B cell; *G-CHOP*, obinutuzumab plus cyclophosphamide, doxorubicin, vincristine, and prednisone; *PFS*, progression-free survival; *OS*, overall survival; *R-CHOP*, rituximab plus cyclophosphamide, doxorubicin, vincristine, and prednisone

Kaplan–Meier analysis of PFS according to treatment arm in patients with different COO subtypes (ABC, GCB, and unclassified) is summarized in Fig. 4. The GCB subgroup appeared to be associated with a better PFS compared with ABC and unclassified subgroups (5-year Kaplan–Meier PFS estimates, 71.0%, 54.3%, and 57.9% in the G-CHOP arm and 65.5%, 55.7%, and 62.7% in the R-CHOP arm; Fig. 4).

No significant reductions in the risk of disease progression with G-CHOP relative to R-CHOP were observed for patients with GCB, ABC, or unclassified DLBCL, although a trend towards benefit with G-CHOP compared to R-CHOP was apparent for the GCB subgroup (stratified HR, GCB 0.80, 95% CI 0.58–1.12; ABC 0.91, 95% CI 0.61–1.36; and unclassified 1.10, 95% CI 0.65–1.88; Fig. 3 and Supplementary Fig 1).

### Safety

Safety results from the final analysis are consistent with those reported in the primary analysis, with no new safety signals. Most patients in each treatment arm (97.6% in the G-CHOP arm and 94.0% in the R-CHOP arm) experienced at least one AE (Table 3). The incidence of grade 3–5 AEs was higher in the G-CHOP arm (75.1%) compared with the R-CHOP arm (65.8%). Serious AEs were also more common in the G-CHOP arm (44.4% vs 38.4%).

**Table 3:** Summary of safety (AEs by preferred term reported by ≥ 5% of patients; safety population)

Variable	R-CHOP (*N* = 701) *n* (%)		G-CHOP (*N* = 702) *n* (%)	
No. of deaths (any reason)	141 (20.1)		149 (21.2)	
No. of patients withdrawn from the study due to an AE	4 (0.6)		6 (0.9)	
Patients with ≥ 1				
AE	659 (94.0)		685 (97.6)	
Grade 3–5 AE	461 (65.8)		527 (75.1)	
AE with fatal outcome^a^	31 (4.4)		43 (6.1)	
Serious AE	269 (38.4)		312 (44.4)	
Treatment-related AE	600 (85.6)		647 (92.2)	
AE leading to withdrawal of any treatment	58 (8.3)		8.7 (12.4)	
AE leading to dose reduction for any treatment	142 (20.3)		145 (20.7)	
	Grade 3–5 AE, *n* (%)	Serious AE, *n* (%)	Grade 3–5 AE, *n* (%)	Serious AE, *n* (%)
Blood and lymphatic system disorders				
Neutropenia	277 (39.5)	38 (5.4)	336 (47.9)	54 (7.7)
Febrile neutropenia	108 (15.4)	71 (10.1)	130 (18.5)	85 (12.1)
Leukopenia	78 (11.1)	–	104 (14.8)	–
Anemia	55 (7.8)	–	53 (7.5)	–
Thrombocytopenia	11 (1.6)	–	40 (5.7)	–
Infections and infestations				
Pneumonia	34 (4.9)	33 (4.7)	44 (6.3)	43 (6.1)

*AE* adverse event, *G-CHOP* obinutuzumab plus cyclophosphamide, doxorubicin, vincristine, and prednisone, *R-CHOP* rituximab plus cyclophosphamide, doxorubicin, vincristine, and prednisone

^a^Fatal AEs that occurred in more than one patient in either group, listed as preferred terms, were as follows: death (cause unknown; 2 patients in the R-CHOP arm and 3 patients in the G-CHOP arm), pneumonia (5 patients in each arm), septic shock (6 patients in the G-CHOP arm), sepsis (3 patients in the R-CHOP arm and 1 patient in the G-CHOP arm), hepatocellular carcinoma (1 patient in the R-CHOP arm and 2 patients in the G-CHOP arm), cerebrovascular accident (2 patients in each arm), and pulmonary embolism (2 patients in the G-CHOP group)

A higher number of patients in the G-CHOP arm compared with the R-CHOP arm discontinued any component of study treatment due to an AE (12.4% vs 8.3%). Fatal AEs occurred in 43 (6.1%) patients in the G-CHOP arm and 31 (4.4%) patients in the R-CHOP arm, with infections being the most common; in particular, five patients (0.7%) in each arm had a fatal case of pneumonia, and six patients (0.9%) in the G-CHOP arm versus no patients in the R-CHOP arm had a fatal case of septic shock (Table 3 and Supplementary Table 1). Overall, the most common cause of death was disease progression (G-CHOP 12.4% and R-CHOP 13.1%).

In total, 22 (3.1%) patients in the G-CHOP arm and 26 (3.7%) patients in the R-CHOP arm had a second malignancy. The most common of these were prostate cancer (0.4% in each arm), lung adenocarcinoma (0.4% and 0.3% in the G-CHOP and R-CHOP arms, respectively), and breast cancer (0.1% and 0.4% in the G-CHOP and R-CHOP arms, respectively). Prolonged neutropenia (0.3% vs 0.0%) and late-onset neutropenia (8.7% vs 4.9%) occurred at a greater frequency in the G-CHOP arm compared with the R-CHOP arm.

## Discussion

This Phase III, open-label, randomized study was designed to compare the efficacy and safety of G-CHOP versus R-CHOP in previously untreated patients with DLBCL. In agreement with the findings of the primary analysis (clinical cut-off date, 29 April 2016) [10], results of this analysis did not demonstrate superiority of G-CHOP compared to R-CHOP.

After a median observation time of 47.7 months, investigator-assessed PFS (study primary endpoint) did not differ significantly between G-CHOP and R-CHOP (stratified HR 0.94, 95% CI 0.78–1.12; *p* = 0.48), with similar results to those obtained in the primary analysis (stratified HR 0.92, 95% CI 0.76–1.11; *p* = 0.39). The results of the secondary endpoints were consistent with the primary endpoint and did not show a benefit of G-CHOP over R-CHOP. The 5-year OS rates were similar for G-CHOP and R-CHOP (77.0% vs 77.7%).

G-chemotherapy has previously demonstrated superiority compared with R-chemotherapy in studies of patients with other B cell malignancies, such as follicular lymphoma (FL) and chronic lymphocytic leukemia [14, 15]. The lack of superior efficacy of G-CHOP compared with R-CHOP observed within this study was unexpected.

In the present study, we noted a trend towards a PFS benefit with G-CHOP versus R-CHOP in patients with GCB DLBCL. Like FL, GCB DLBCL is derived from germinal center B cells, and many driver genetic alterations that provide a selective advantage and contribute to cancer development are seen in both lymphomas [16]. This may, in part, explain the trend in benefit observed in the patients with GCB DLBCL, which is consistent with the improvement in outcome seen in patients with FL when treated with G-CHOP versus R-CHOP [14]. Interestingly, we found that the ABC DLBCL subgroup was associated with better PFS (5-year Kaplan–Meier PFS estimates: G-CHOP 54.3% and R-CHOP 55.7%) than reported in retrospective population analyses of previously untreated patients with DLBCL treated with R-CHOP (5-year Kaplan–Meier PFS estimates: R-CHOP 46% [17] and 48% [18]). This result may indicate a selection bias for low-risk patients in the GOYA trial, with very high-risk patients requiring urgent treatment less likely to be enrolled into a prospective trial such as this. It is noteworthy that the total median time from diagnosis to randomization was 24.0 days (range, 1.0–1104.9). While it is unclear how selection bias may have affected the overall result, there was no significant improvement in outcome with G-CHOP in any IPI subgroup.

The frequency and nature of the AEs reported were as expected for the patient population and the treatment regimens being assessed, and no new safety signals were observed during the additional follow-up after the primary analysis. Higher rates of grades 3–5 and serious AEs occurred in the G-CHOP arm compared with the R-CHOP arm; however, these were generally clinically manageable. A similar percentage of deaths occurred in the G-CHOP and R-CHOP arms (21.2% vs 20.1%), and the majority of these fatalities were due to disease progression (12.4% vs 13.1%). There were no unexpected delayed toxicities observed, and a similar number of second malignancies occurred in the G-CHOP and R-CHOP arms (3.1 and 3.7%, respectively). A higher frequency of late-onset neutropenia was observed in the G-CHOP arm compared with the R-CHOP arm (8.7% vs 4.9%, respectively).

## Conclusions

In conclusion, the final analysis of this study demonstrated that G-CHOP did not show a PFS benefit over R-CHOP in previously untreated patients with DLBCL, and R-CHOP remains the standard of care in this population. The results of the secondary endpoints were consistent with the primary endpoint. Overall, no unexpected safety findings were observed, and the toxicity of G-CHOP was generally manageable. Exploratory analyses and investigations of biomarkers are ongoing to evaluate whether there may be a role for G in identifiable subgroups of DLBCL.

## Supplementary information

**Additional file 1: Supplementary Table 1**. Grade 5 (fatal) adverse events (safety evaluable population). **Supplementary Fig. 1.** Kaplan–Meier estimates of PFS by treatment group for COO subtypes. **A** Investigator-assessed PFS by treatment arm in the GCB subgroup, in which a trend towards a better PFS with G-CHOP was observed; **B** Investigator-assessed PFS by treatment arm in the ABC subgroup, where no difference in PFS between treatment arms was observed; **C** Investigator-assessed PFS by treatment arm in the unclassified subgroup, in which there was also no difference in PFS between treatment arms.

## Data Availability

Qualified researchers may request access to individual patient level data through the clinical study data request platform (https://vivli.org/). Further details on Roche's criteria for eligible studies are available here (https://vivli.org/members/ourmembers/). For further details on Roche's Global Policy on the Sharing of Clinical Information and how to request access to related clinical study documents, see here (https://www.roche.com/research_and_development/who_we_are_how_we_work/clinical_trials/our_commitment_to_data_sharing.htm).

## Abbreviations

ABC: Activated B cell
AE: Adverse event
CHOP: Cyclophosphamide, doxorubicin, vincristine, and prednisone
CI: Confidence interval
COO: Cell of origin
CR: Complete response
DFS: Disease-free survival
DLBCL: Diffuse large B cell lymphoma
ECOG: Eastern Cooperative Oncology Group
EFS: Event-free survival
FL: Follicular lymphoma
GCB: Germinal center B cell
G: Obinutuzumab
G-CHOP: Obinutuzumab plus cyclophosphamide, doxorubicin, vincristine, and prednisone
G-CSF: Granulocyte-colony stimulating factor
HR: Hazard ratio
IPI: International Prognostic Index
IRC: Independent review committee
IV: Intravenous
KM: Kaplan–Meier
ORR: Overall response rate
OS: Overall survival
PET: Positron emission tomography
PFS: Progression-free survival
R: Rituximab
R-CHOP: Rituximab plus cyclophosphamide, doxorubicin, vincristine, and prednisone
