# Supplementary material for: A randomized, open-label, Phase III study of obinutuzumab or rituximab plus CHOP in patients with previously untreated diffuse large B-Cell lymphoma: final analysis of GOYA
Source: J Hematol Oncol. 2020 Jun 6;13:71. doi: 10.1186/s13045-020-00900-7 (PMC7276080; doi:10.1186/s13045-020-00900-7)
Supplement: Supplementary file 1 — Additional file 1: Supplementary Table 1. Grade 5 (fatal) adverse events (safety evaluable population). Supplementary Fig. 1. Kaplan–Meier estimates of PFS by treatment group for COO subtypes. A Investigator-assessed PFS by treatment arm in the GCB subgroup, in which a trend towards a better PFS with G-CHOP was observed; B Investigator-assessed PFS by treatment arm in the ABC subgroup, where no difference in PFS between treatment arms was observed; C Investigator-assessed PFS by treatment arm in the unclassified subgroup, in which there was also no difference in PFS between treatment arms. [file 13045_2020_900_MOESM1_ESM.docx]

**Supplementary appendix**

**Supplementary Table 1:** Grade 5 (fatal) adverse events (safety evaluable population).

| Adverse event | R-CHOP (N=701),  n (%) | G-CHOP (N=702),  n (%) |
| --- | --- | --- |
| **Number of patients with ≥1 event** | **31 (4.4)** | **43 (6.1)** |
| ***Blood and lymphatic system disorder*** | ***0*** | ***2 (0.3)*** |
| Febrile neutropenia | 0 | 1 (0.1) |
| Histiocytosis hematophagic | 0 | 1 (0.1) |
| ***Cardiac disorders*** | ***3 (0.4)*** | ***4 (0.6)*** |
| Cardiac arrest | 1 (0.1) | 1 (0.1) |
| Cardiac failure | 1 (0.1) | 0 |
| Cardiac failure congestive | 0 | 1 (0.1) |
| Cardiopulmonary failure | 1 (0.1) | 0 |
| Coronary artery thrombosis | 0 | 1 (0.1) |
| Myocardial infarction | 0 | 1 (0.1) |
| ***Gastrointestinal disorders*** | ***4 (0.6)*** | ***1 (0.1)*** |
| Gastric hemorrhage | 1 (0.1) | 0 |
| Gastrointestinal hemorrhage | 1 (0.1) | 0 |
| Impaired gastric emptying | 1 (0.1) | 0 |
| Intestinal ischemia | 0 | 1 (0.1) |
| Intestinal obstruction | 1 (0.1) | 0 |
| Intestinal perforation | 1 (0.1) | 0 |
| ***General disorders and administration site conditions*** | ***3 (0.4)*** | ***5 (0.7)*** |
| Death | 2 (0.3) | 3 (0.4) |
| Sudden death | 1 (0.1) | 1 (0.1) |
| General physical health deterioration | 0 | 1 (0.1) |
| ***Infections and infestations*** | ***11 (1.6)*** | ***16 (2.3)*** |
| Pneumonia | 5 (0.7) | 5 (0.7) |
| Septic shock | 0 | 6 (0.9) |
| Sepsis | 3 (0.4) | 1 (0.1) |
| Bronchopulmonary aspergillosis | 1 (0.1) | 1 (0.1) |
| Candida sepsis | 1 (0.1) | 0 |
| Escherichia sepsis | 1 (0.1) | 0 |
| Infection | 0 | 1 (0.1) |
| Meningitis viral | 0 | 1 (0.1) |
| Osteomyelitis | 0 | 1 (0.1) |
| Peritonitis | 1 (0.1) | 0 |
| ***Injury, poisoning and procedural complications*** | ***1 (0.1)*** | ***2 (0.3)*** |
| Abdominal wound dehiscence | 1 (0.1) | 0 |
| Subarachnoid hemorrhage | 0 | 1 (0.1) |
| Subdural hematoma | 0 | 1 (0.1) |
| ***Metabolism and nutrition disorders*** | ***1 (0.1)*** | ***0*** |
| Failure to thrive | 1 (0.1) | 0 |
| ***Neoplasm benign, malignant and unspecified (including cysts and polyps)*** | ***5 (0.7)*** | ***5 (0.7)*** |
| Hepatocellular carcinoma | 1 (0.1) | 2 (0.3) |
| Acute myeloid leukemia | 1 (0.1) | 0 |
| Breast cancer | 0 | 1 (0.1) |
| Colon cancer | 1 (0.1) | 0 |
| Lung adenocarcinoma | 1 (0.1) | 0 |
| Lung neoplasm malignant | 0 | 1 (0.1) |
| Myelodysplastic syndrome | 0 | 1 (0.1) |
| Squamous cell carcinoma of pharynx | 1 (0.1) | 0 |
| ***Nervous system disorders*** | ***3 (0.4)*** | ***2 (0.3)*** |
| Cerebrovascular accident | 2 (0.3) | 2 (0.3) |
| Toxic encephalopathy | 1 (0.1) | 0 |
| ***Psychiatric disorders*** | ***0*** | ***1 (0.1)*** |
| Completed suicide | 0 | 1 (0.1) |
| ***Respiratory, thoracic and mediastinal disorders*** | ***3 (0.4)*** | ***6 (0.9)*** |
| Acute respiratory failure | 1 (0.1) | 1 (0.1) |
| Pulmonary embolism | 0 | 2 (0.3) |
| Acute respiratory distress syndrome | 0 | 1 (0.1) |
| Dyspnea | 0 | 1 (0.1) |
| Interstitial lung disease | 1 (0.1) | 0 |
| Pulmonary edema | 0 | 1 (0.1) |
| Respiratory failure | 1 (0.1) | 0 |
| ***Vascular disorders*** | ***0*** | ***1 (0.1)*** |
| Hemorrhage | 0 | 1 (0.1) |

*G-CHOP, obinutuzumab plus cyclophosphamide, doxorubicin, vincristine, and prednisone; R-CHOP, rituximab plus cyclophosphamide, doxorubicin, vincristine, and prednisone.*

**Supplementary Fig. 1** Kaplan–Meier estimates of PFS by treatment group for COO subtypes.

**A** Investigator-assessed PFS by treatment arm in the GCB subgroup, in which a trend towards a better PFS with G-CHOP was observed;

**B** Investigator-assessed PFS by treatment arm in the ABC subgroup, where no difference in PFS between treatment arms was observed;

**C** Investigator-assessed PFS by treatment arm in the unclassified subgroup, in which there was also no difference in PFS between treatment arms.

| 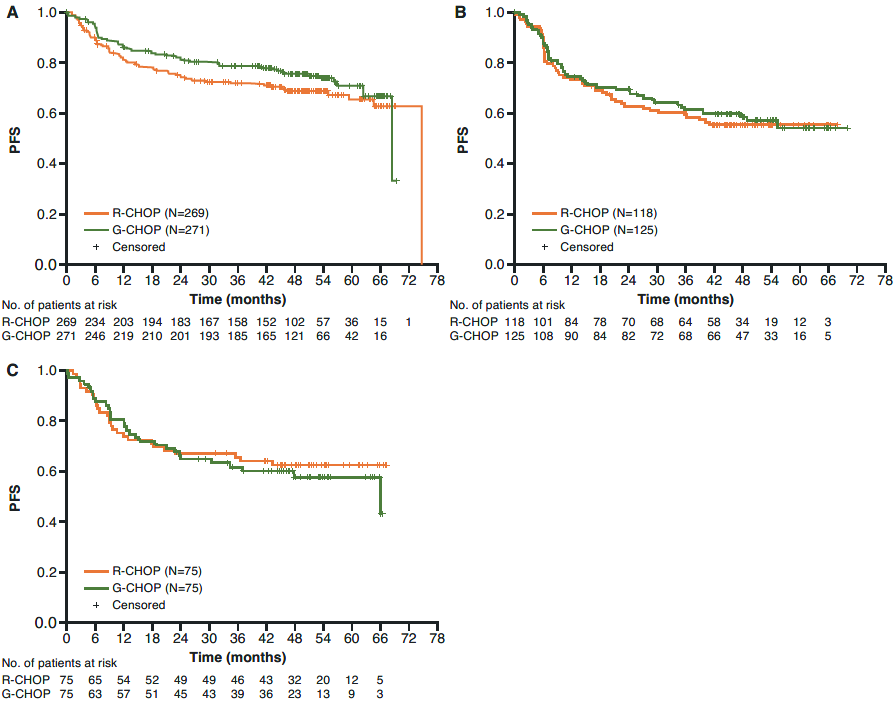 |
| --- |

*ABC, activated B cell; COO, cell of origin; GCB, germinal center B cell; G-CHOP, obinutuzumab plus cyclophosphamide, doxorubicin, vincristine, and prednisone; PFS, progression-free survival; R-CHOP, rituximab plus cyclophosphamide, doxorubicin, vincristine, and prednisone.*
